# A common haplotype within the PON1 promoter region is associated with sporadic ALS

**DOI:** 10.1080/17482960802233177

**Published:** 2009-07-10

**Authors:** John E. Landers, Lijia Shi, Ting-Jan Cho, Jonathan D. Glass, Christopher E. Shaw, P. Nigel Leigh, Frank Diekstra, Meraida Polak, Ildefonso Rodriguez-Leyva, Stephan Niemann, Bryan J. Traynor, Diane Mckenna-Yasek, Peter C. Sapp, Ammar Al-Chalabi, Anne-Marie A. Wills, Robert H. Brown

**Affiliations:** ^a^ Cecil B Day Laboratory for Neuromuscular Research, Massachusetts General Hospital East, , Charlestown, MA, USA; ^b^ Department of Neurology, MRC Centre for Neurodegeneration Research, King's College London, Institute of Psychiatry, London, UK; ^c^ Center for Neurodegenerative Disease, School of Medicine, Emory University, , Atlanta, Georgia, USA; ^d^ Department of Neurology, Hospital Central, Colonia Universitaria, San Luis Potosi, Mexico

**Keywords:** Amyotrophic lateral sclerosis, paraoxonase, SNP, haplotypes, case-control studies

## Abstract

Amyotrophic lateral sclerosis (ALS) is a progressive, neurodegenerative disorder of upper and lower motor neurons. Genetic variants in the paraoxonase gene cluster have been associated with susceptibility to sporadic ALS. Because these studies have yielded conflicting results, we have further investigated this association in a larger data set. Twenty SNPs spanning the paraoxonase gene cluster were genotyped on a panel of 597 case and 692 control samples and tested for association with risk of sporadic ALS and with ALS sub-phenotypes. Our study revealed two SNPs, rs987539 and rs2074351, within the paraoxonase gene cluster that are associated with susceptibility to sporadic ALS (uncorrected *p*=6.47E-04 and 7.87E-04, respectively). None of the 20 SNPs displayed significant associations with age of onset, site of onset or disease survival. Using a sliding window approach, we have also identified a 5-SNP haplotype that is significantly associated with risk of sporadic ALS (*p*=2.75E-05). We conclude that a common haplotype within the PON1 promoter region is associated with susceptibility to sporadic ALS.

## Introduction

ALS is an incurable, late-onset disease in which motor neurons deteriorate leading to muscular atrophy, weakness and death due to respiratory failure [1]. ALS typically develops in the fifth decade of life and is fatal within 3–5 years [2]. Familial ALS (FALS) accounts for 10% of all ALS cases [3]. Approximately 20% of FALS are due to mutations in the SOD1 gene and other genes [4–9]. Additional loci contributing to FALS have also been identified on chromosomes 9, 16, and 20 [10–13]. In contrast, 90% of ALS cases are sporadic in nature (SALS). Little is known about the factors contributing to the development of SALS. Twin studies have shown that the heritability of SALS is between 0.38 and 0.85 [14], indicating that there is likely to be a significant contribution of non-genetic factors, such as exposure to adverse environmental agents. Based on this evidence, we postulated that environmental response genes might influence the risk of developing SALS. The human paraoxonases represent such an environmental response activity.

The paraoxonase gene cluster consists of three genes (cen-PON1-PON3-PON2-tel) located adjacent to each other in a region spanning ˜140 kb on chromosome 7q21.3-q22.1 [15]. Several lines of evidence indicate that this cluster may influence the risk of developing SALS. First, all three PON enzymes contain lipid antioxidant properties [16], [17]. Heightened oxidative pathology has been implicated in both SALS and FALS [18], although its origin and significance are debated. Secondly, PON1 also functions to detoxify several organophosphate compounds, many of which are neurotoxins found in insecticides, nerve gases, foods, and other household items [16], [17]. Thirdly, genetic variants in the PON genes have also been linked to enhanced risk for other neurological disorders such as Alzheimer's disease [19], [20], Parkinson's disease [21], [22] and dementia [23], as well as atherosclerosis [24]. Finally, four recent studies have described an association between the PON gene cluster and SALS [25–28], although the conclusions of these studies are not entirely consistent [29]. Each study observed a different association peak: two were within differing PON1 amino acid variants [25], [28], one was within the PON1 promoter region [26] and one was within a PON3/PON2 haplotype [27].

To further explore the hypothesis that paraoxonase is related to the risk of SALS, we have tested the potential association of multiple SNPs spanning the PON gene cluster with both ALS susceptibility and phenotypes. Our results confirm the observation that SNPs within this cluster are indeed over-represented in ALS cases compared to controls.

## Methods and materials

### Study subjects

All sporadic ALS patients fulfilled El Escorial criteria [30] for probable or definite ALS. All subjects (including controls) were self-reported Caucasians from the United States. Informed consent was obtained from all individuals in accordance with the requirements of the participating institutional review boards. Age of onset and site of onset information were required for all samples. Duration information was known for 410 deceased cases. A portion of the control DNA samples was purchased from Coriell Cell Repositories. Whole blood from anonymous individuals used for arylesterase assays was purchased from Innovative Research (Southfield, MI). The blood was isolated from apparently healthy individuals ranging in ages 40–64 years (average 47.2 years). The ethnic background of the samples was 63.5% African American, 31.7% Caucasian, and 4.8% Latino. The gender of the samples was 67.3% male.

### Tag SNP selection

Tag SNPs from the PON cluster were selected using an algorithm based on the *r* ^2^ linkage disequilibrium (LD) statistic [31]. Selection of SNPs was facilitated through the use of the software SNPbrowser v. 2.0 using a pairwise *r* ^2^ value of > = 0.99 within the Caucasian population. The PON cluster was defined as the region 10 kb downstream of the last exon of PON1 to 10 kb downstream of the last exon of PON2.

### SNP genotyping

All genotyping was performed using a 5’ Nuclease Assay (TaqMan). Reactions volumes were 5 ul and performed in 384-well format. Each reaction consisted of either genomic or whole genome amplified DNA (5 ng/reaction), 1X TaqMan Universal PCR Master Mix, No AmpErase UNG and 1X Validated TaqMan SNP Assay probes (Applied Biosystems). Reactions were thermocycled with an initial denaturation step of 95^o^C for 10 min, followed by 50 cycles of 95°C for 15 s and 60^o^C for 1 min. The assay results were collected using an ABI 7900HT Real-time PCR instrument and genotyping calls were performed using SDS 2.0 software (Applied Biosystems).

### Allelic association and linkage disequilibrium analyses

Allelic association testing and inferring individual haplotypes was performed using the software application PLINK v0.99p [32] (http://pngu.mgh.harvard.edu/˜purcell/plink/). Haploview v4.0 (http://www.broad.mit.edu/mpg/haploview/) was used to determine the linkage disequilibrium in the PON region. Bonferroni multiple test correction was applied by multiplying PLINK-derived *p*-values by the number of SNPs or haplotypes assayed.

### Serum paraoxonase activity levels

The levels of paraoxanase activity in human serum were determined using an arylesterase/paraoxonase assay kit (Zeptometerix). Briefly, arylesterase substrate buffer (20 mM Tris HCl, pH 8.0, 1 mM CaCl_2_, 4 mM phenyl acetate) was added to 2 ul serum within triplicate wells of a 96-well UV transparent microtiter plate. The rate of hydrolysis of phenyl acetate to phenol formation was monitored by measuring the absorbance at 260 nm at 25^o^C. Data points were collected every 45 s for a total of six time points. The change in absorbance per minute was calculated and arylesterase activity was determined by comparing to known standards and adjusting for serum protein concentration as determined by a Bradford assay. Association of serum levels to genotypes and haplotypes was performed using a linear regression model adjusting for age, race and gender. Power calculations utilized standard deviations of arylesterase activity previously determined [33]. The non-parametric Wilcoxon two-sided test and Kruskall-Wallis test were used for race-stratified association analysis of haplotypes and genotypes, respectively.

## Results

### Sample selection

To investigate the role of PON genes in sporadic ALS, 692 case and 597 control samples were selected for genotyping. All samples chosen were Caucasian to prevent false-positives due to ethnic-specific polymorphisms. Table I provides a detailed breakdown of the samples used in this study.

**Table I.** Distribution of sporadic ALS cases and control.

		Total	Males	Females	Age±SD
Controls	Total	692	426	266	59.64±14.40
Cases	Total	597	390	207	53.92±13.05
	Bulbar	141	79	62	57.41±12.88
	Upper limb	237	190	47	51.05±13.08
	Lower limb	184	94	90	54.25±12.47
	Multiple/Other	35	27	8	57.50±12.45

### Allelic association of the PON genes to sporadic ALS

Tag SNPs were selected from the PON gene cluster based on the *r* ^2^ linkage disequilibrium (LD) statistic. To increase the power of our study, selection was performed at a conservative level of *r* ^2^=0.99. Eighteen SNPs were selected over the PON cluster region. In addition, three additional SNPs were selected because of their previously established functional significance in the PON1 gene. A glutamine (Q) to arginine (R) polymorphism (rs662) located at codon 192 (Q192R) has been shown to alter PON1 activity; the R isoform is more efficient at detoxifying paraoxon whereas the Q isoform is more efficient at detoxifying sarin and soman [34]. Additionally, a L55M polymorphism (rs854560) influences PON1 serum levels with the M variant displaying lower plasma levels [34]. PON1 serum levels are also influenced by a promoter polymorphism located at position A–161G (rs705381) [35].

Each SNP was genotyped in the panel cases and controls. One SNP was subsequently removed from further analysis because of a low call rate. Allelic association analysis was performed for each SNP and a Bonferroni multiple test correction was applied. The results of the analysis are shown in Table II. Two SNPs within the PON cluster displayed significant association after correction. SNP #16 (rs987539), located within intron 6 of PON2, was most significantly associated (*p = *6.47E-04). SNP #6 (rs2074351), located ˜90 bp upstream of the start of the exon 2 of PON1, displayed the second most significant association (*p = *7.87E-04). Interestingly, the R192Q, L55M and A–161G polymorphisms, represented by SNPs #4, #5 and #9, respectively, did not show significant association after multiple test correction (Table II).

**Table II.** Association testing of PON cluster variants with sporadic ALS. Bold text represents SNPs with corrected *p* <0.05. HWE represents the Hardy-Weinberg test statistics for each SNP using all samples.

	SNP ID	Case Freq.	Cont. Freq.	Alleles	*p*-value	Adj. *p*-value	χ^2^	OR	HWE
1	rs854543	0.208	0.194	A > C	0.39230	1	0.73	1.09	0.140
2	rs854549	0.334	0.361	C > A	0.14720	1	2.10	0.89	0.539
3	rs2237582	0.322	0.279	A > G	0.01952	0.390	5.45	1.23	0.422
4	rs662	0.318	0.279	T > C	0.03765	0.753	4.32	1.20	0.378
5	rs854560	0.351	0.369	A > T	0.33030	1	0.95	0.92	0.542
6	rs2074351	0.324	0.263	G > A	7.87E-04	0.016	11.27	1.34	0.274
7	rs854565	0.280	0.317	G > A	0.04440	0.888	4.04	0.84	0.893
8	rs2299261	0.337	0.381	A > G	0.02325	0.465	5.15	0.83	0.363
9	rs705381	0.221	0.261	C > T	0.01856	0.371	5.54	0.80	0.939
10	rs705382	0.343	0.393	G > C	0.00863	0.173	6.90	0.81	0.048
11	rs4141217	0.486	0.442	C > T	0.02590	0.518	4.96	1.20	0.612
12	rs916864	0.212	0.171	C > T	0.00937	0.187	6.75	1.30	0.928
13	rs3757708	0.477	0.437	T > G	0.04077	0.815	4.19	1.18	0.778
14	rs10487132	0.407	0.427	A > G	0.30230	1	1.06	0.92	0.688
15	rs2072200	0.218	0.174	G > C	0.00528	0.106	7.78	1.32	1.000
16	rs987539	0.488	0.421	C > T	6.47E-04	0.013	11.64	1.31	0.398
17	rs2286233	0.115	0.129	A > T	0.25840	1	1.28	0.87	0.439
18	rs11981433	0.402	0.444	T > C	0.03406	0.681	4.49	0.84	0.568
19	rs43037	0.391	0.421	T > C	0.12290	1	2.38	0.88	0.417
20	rs10953147	0.478	0.432	A > G	0.01902	0.380	5.50	1.21	0.911

### Phenotypic association analysis

We also investigated the possibility that polymorphisms within the paraoxonase gene cluster are associated with ALS sub-phenotypes. Each of the 20 SNPs was tested for association with age of onset, site of onset (bulbar vs. spinal and upper limb vs. lower limb) and disease survival. The results from this analysis are shown in Table III. Although four *p*-values were observed below 0.05, none was significant after Bonferroni multiple test correction. We therefore conclude that the paraoxonase gene cluster is not associated with age of onset, site of onset, or survival within sporadic ALS.

**Table III.** Uncorrected *p*-values for association with ALS sub-phenotypes.

	SNP ID	Onset age	Bulbar vs. spinal	Upper vs. lower limb	Survival
1	rs854543	0.4581	0.2510	0.3297	0.1912
2	rs854549	0.4581	0.2177	0.0808	0.9886
3	rs2237582	0.8156	0.9216	0.6212	0.0072
4	rs662	0.7722	0.9619	0.5685	0.0100
5	rs854560	0.7694	0.0136	0.1073	0.6424
6	rs2074351	0.7453	0.1251	0.4560	0.5906
7	rs854565	0.4559	0.2188	0.1903	0.3044
8	rs2299261	0.2343	0.0645	0.8742	0.7488
9	rs705381	0.4274	0.1799	0.0732	0.3185
10	rs705382	0.5799	0.0811	0.2236	0.9660
11	rs4141217	0.6472	0.6720	0.6039	0.4512
12	rs916864	0.8488	0.5769	0.5047	0.0865
13	rs3757708	0.7117	0.5384	0.5639	0.3483
14	rs10487132	0.6322	0.3119	0.4016	0.3968
15	rs2072200	0.8045	0.6148	0.5548	0.0398
16	rs987539	0.7564	0.6757	0.8730	0.2237
17	rs2286233	0.5399	0.0637	0.6141	0.7118
18	rs11981433	0.5467	0.5181	0.8226	0.2929
19	rs43037	0.5219	0.8138	0.6530	0.5452
20	rs10953147	0.1362	0.7346	0.4733	0.2579

### Linkage disequilibrium within the PON cluster

**Figure 1.**
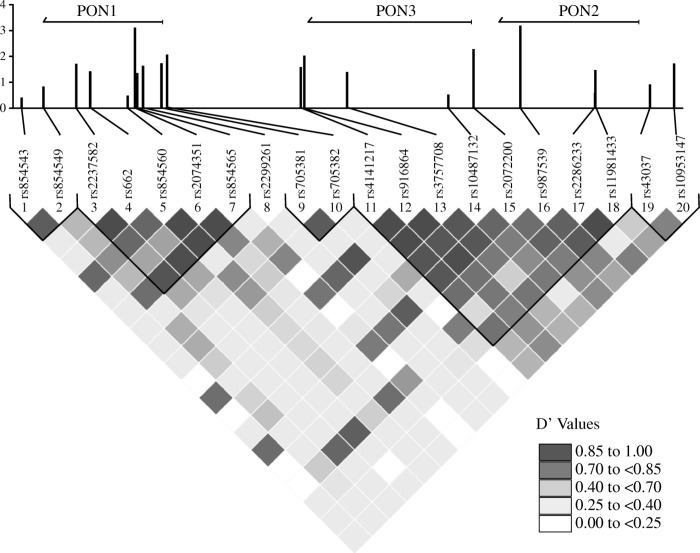
Linkage disequilibrium plot for SNPs within the PON cluster. Pairwise linkage disequilibrium values (D’) were calculated for SNPs spanning the PON cluster. The color key for D’ values is shown. The –log_10_(*p*) values for association with risk are shown above each SNP. The location of the three paraoxonase genes and their genomic position on chromosome 7 is shown above the plot. Diagonal lines indicate the linkage disequilibrium blocks.

The foregoing association studies suggest that the PON1 and/or PON2 gene is associated with SALS. It is possible that this association is not due to the effects of the PON1 or PON2 gene per se but rather reflects linkage disequilibrium of the associated SNPs with another gene. To test this possibility, we investigated the extent of linkage disequilibrium within the PON region. Genotyping results were used to determine the linkage disequilibrium between each pair of SNPs within the PON cluster as shown in Figure 1. These results demonstrate that the PON cluster appears to be broken into smaller blocks of linkage disequilibrium. In particular, rs987539, which displayed the highest level of association, is present within linkage disequilibrium block consisting of SNPs #11–18, which extends beyond the 3’ end of PON3 and possibly to the promoter region of PON1. Furthermore, rs2074351, which displayed the second highest level of association, is present within linkage disequilibrium block consisting of SNPs #3–7, which are all present within the PON1 gene.

### Haplotypic association analysis

To further investigate the association of the PON genes with SALS, we considered the possibility that a stronger association may be observed by establishing the association of haplotypes within the PON region. Towards this end, we measured the association of 5-SNP haplotypes in a moving window across the PON cluster to SALS. This analysis (Table IV) revealed a 5-SNP haplotype, consisting of SNPs #7–11 (rs854565, rs2299261, rs705381, rs705382, and rs4141217), that displayed a strong association with SALS (*p = *2.75E-05). The next lowest observed *p*-value is over nine times higher and corresponds to the overlapping previous 5-SNP window (SNP #6–10). The genomic region of the 5-SNP haplotype, termed HAP1, spans intron 1 of the PON1 gene to approximately 30 kb upstream of the start of the PON1 gene (Figure 1).

**Table IV.** Top haplotype within the PON cluster associated with sporadic ALS. The haplotype shown consists of SNPs rs854565, rs2299261, rs705381, rs705382 and rs4141217.

Haplotype	Case Freq.	Cont. Freq.	*p*-value	Adjusted *p*-value	χ^2^	OR
GACGT	0.2546	0.1849	2.75E-05	3.86E-03	17.58	1.38

### Serum paraoxonase activity levels and PON genotypes/haplotypes

**Figure 2.**
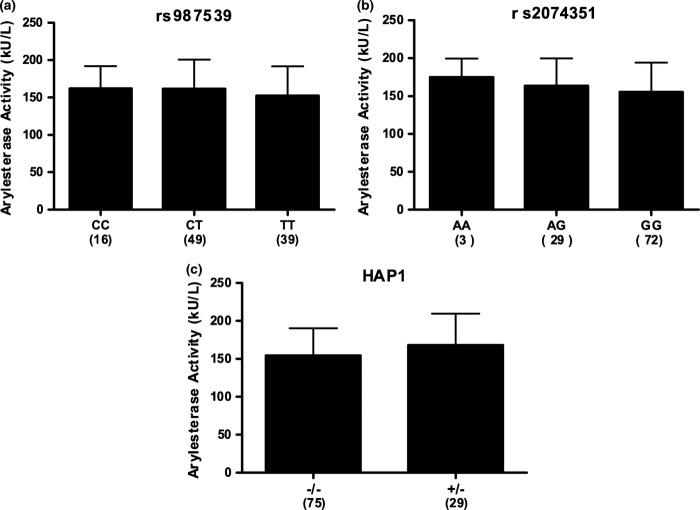
PON1 arylesterase activity in control serum. PON1 arylesterase activity was determined for 104 healthy control individuals. DNA isolated from the same samples was genotyped. The graphs represent the arylesterase activity observed for (a) rs987539 genotypes, (b) rs2074351 genotypes, and (c) the SALS-associated HAP1 haplotype. No individuals were observed that were homozygous for the HAP1 haplotypes. The numbers in parenthesis represent the sample size for the group shown. Error bars represent the standard deviation of each group.

Based on the location of the associated 5-SNP haplotype, it is reasonable to hypothesize that the expression of PON1 is altered in individuals harboring HAP1. To investigate this possibility, we compared the PON1 expression levels of individuals to their respective haplotypes. Human blood was collected from 104 healthy control individuals. From each sample, one aliquot was used to isolate DNA, while another provided a serum sample. The DNA was genotyped for the 5-SNPs that compose the SALS-associated haplotype, as well as rs2074351 and rs987539, the SNPs displaying significant association. The arylesterase activity was measured in each serum sample using phenyl acetate as the substrate for hydrolysis. Previous studies have shown that the arylesterase activity directly reflects the protein levels of PON1 in serum [35], [36]. Haplotypes using the 5-SNPs that composed HAP1 were inferred using the software application PLINK and compared to the arylsterase activity (Figure 2). No individuals were observed to be homozygous for the HAP1 haplotype. Using a linear regression model, no significant association was observed between the HAP1 haplotype and arylesterase activity (*p = *0.587). Statistical calculations indicate that we have 94% power to detect a 20% difference and a 79% power to detect a 15% difference in arylesterase levels. Similarly, we also compared the arylesterase activity observed for individuals harboring each of the three genotypes for the two significantly associated SNPs (Figure 2). Although slight differences are observed between each genotype for each of these markers, neither rs987539 (*p = *0.208) nor rs2074351 (*p = *0.184) were significantly different. Due to the fact that the serum samples were derived from an ethnically mixed population, we also attempted to determine association after stratifying by race. However, testing of HAP1, rs2074351, and rs987539 still failed to reveal a significant association with either African-Americans (*p = *0.226, 0.0962, 0.1462, respectively) or Caucasians (*p = *0.986, 0.907, 0.571, respectively). Based on these results, we cannot conclude that the rs987539/rs2074351 genotypes or HAP1 haplotypes modify PON1 arylesterase activity levels, as determined by phenyl acetate hydrolysis.

## Discussion

This study confirms previous reports that genetic variants within the PON gene cluster are associated with susceptibility to SALS. This association prompts consideration of the biological function of PON1 and mechanisms by which its variants may predispose to SALS. PON1 detoxifies several neurotoxic organophosphate compounds often found in insecticides, nerve gas, foods, and other household items [16], [17]. It therefore seemed reasonable at the outset of this study to postulate that decreased activity of PON1 increases risk with exposure to injurious neurotoxins, leading over time to SALS. This concept is consistent with studies showing that SALS is not fully explained by genetic factors (heritability = 0.38–0.85) [14], and by the epidemiological observations that ALS cases have an increased exposure to insecticides and pesticides and that some occupations confer a higher ALS risk (e.g. farmers [37], members of the military in general and military personnel who were deployed in the first Gulf War [38], [39]). If indeed PON1 influences SALS susceptibility through capacity to detoxify specific toxins, then the identification of its environmental substrates as well as factors increasing the expression/activity of PON1 may illuminate aspects of the pathogenesis of SALS and ultimately be helpful in treating or reducing the risk of this disease.

Although the current study confirms the association of PON1 with sporadic ALS, it did not document a correlation between the disease-associated haplotype HAP1 and levels of PON1 activity, as measured by phenyl acetate hydrolysis. This parallels our observation reported elsewhere that ALS sera also do not show reduced hydrolysis of paraoxon, diazoxon or phenyl acetate compared to controls [33]. These findings raise the possibility that the ALS-related genetic variants in PON1 alter activity towards some substrate other than phenyl acetate or paraxon. Indeed, PON1 is highly promiscuous, hydrolyzing hundreds of substrates. Moreover, it is highly polymorphic with more than 160 known polymorphisms, many of which differentially affect hydrolysis of different substrates [40].

We also note that PON1 and PON2 are potent inhibitors of lipid oxidation; PON1 is tightly associated with HDL particles and is substantially anti-atherogenic [41–43]. Therefore variations in the lipid anti-oxidant properties of ALS-associated PON1 and PON2 polymorphisms may also contribute to SALS susceptibility. There are several lines of inquiry incriminating oxidative cytotoxicity as a causative factor in ALS, although this remains controversial. By analogy, heightened oxidative toxicity is reported in other neurodegenerative disorders (e.g. Alzheimer's and Parkinson's disease [44]). Reports have also shown that the risk of SALS can be reduced by the intake of antioxidants [45]. It is possible that detoxification and antioxidant properties both contribute to influencing the susceptibility of SALS. The study of PON1 variants that have lost their antioxidant properties, but not their detoxification properties, may be useful in dissecting this question.

This is the fifth report implicating variants in paraoxonases as susceptibility factors for SALS [25–28]. It is potentially of importance that there are inconsistencies in these studies. Each study observed a different association peak; two studies observed association peaks at differing PON1 amino acid variants, L55M and Q192R [25], [28], neither which were significant in our study. One study observed an association within the PON1 promoter region [26] and one was within a PON3/PON2 haplotype [27]. Thus, three of the previous reports implicate PON1 as an ALS risk factor while the fourth implicates PON2 and 3. Furthermore, even among the reports associating PON1 with SALS there is no consistency in the associated variants. At least three factors may explain this. First, the SALS patients were derived from different populations in the studies (Poland, Australia, Ireland, North American Caucasians, US Caucasians). Conceivably, linkage disequilibrium patterns within the PON cluster may differ in each population, leading to divergent results. Variations in the patterns of linkage disequilibrium, as well as the level of association with a particular phenotype, are common among different populations. Such variations have been shown both on the genomic level [46], [47] and within the PON cluster itself [20]. Secondly, it is possible that more than one variant in the PON1 gene can increase SALS susceptibility; differing observations may reflect the frequencies of the causal variants in that given population. Thirdly, each population may differ in the patterns of exposure to environmental toxins and to compounds that influence paraoxonase expression. For example, smoking [48], diet [49], lipid-controlling medication exposure [50] and organophosphate exposure [51] (both amount and type) are all known to modify levels of PON1 expression; each is likely to vary among different populations. Of note, no interaction was seen between self-reported pesticide exposure and PON1 genetic variants in a small study by Morohan et al. [26] We propose future studies of PON1 that would test for interactions between the above environmental exposures with the genotypes of interest.

To date, there have been five publications that use a whole genome association (WGA) approach to identify risk factors for sporadic ALS [47], [52–55]. However, none of the studies has identified the PON cluster as such a risk factor. Several factors could contribute to the difference in observations between the WGA studies and this study. First, all of the WGA studies have utilized genotyping products, from either Illumina or Affymetrix, which do not contain the two significant SNPs identified within this study (rs2074351 and rs987539). As such, a significant association would be based on SNPs within the panel that are in high linkage disequilibrium with rs2074351 or rs987539. Additionally, these studies, due to the large number of SNPs genotyped, utilize an approach of further testing those variants below a cut-off *p*-value within a confirmatory population set. In most cases, the cut-off *p-*value assigned (typically *p* <1E-04) within the WGA studies would not have included the significant SNPs within this study (*p = *6.47E-04 and 7.87E-04). Furthermore, three of the WGA studies [52], [54], [55] utilized populations collected from Europe (The Netherlands or Ireland). It is conceivable that the association results observed in this study may not be applicable to other populations. Furthermore, the size of the population described within this study is larger than those utilized in nearly all of the WGA studies. As a result, the WGA studies may be underpowered to detect an association with the PON cluster. Finally, all association studies are subject to the randomness of the population collected and power to detect the association. Therefore, the inability to detect association may just reflect such inherent differences in the populations tested.

## Acknowledgements

The Day Neuromuscular Research Laboratory (RHB) receives support from the National Institute of Neurological Disease and Stroke, the National Institute for Aging, the Angel Fund, the ALS Association, Project ALS, the Pierre L. de Bourgknect ALS Research Foundation and the Al-Athel ALS Foundation. RHB and JEL received support for this project from the ALS Therapy Alliance. JDG receives support from the Packard Center for ALS Research. AA-C is supported by the Medical Research Council, Motor Neurone Disease Association, ALS Association and Project ALS. PS is supported through the auspices of H. R. Horvitz, an Investigator of the Howard Hughes Medical Institute, Department of Biology, Massachusetts Institute of Technology. A.-M.W. is supported by the American Academy of Neurology Foundation and ALS Association. ***Declaration of interest:*** The authors report no conflicts of interest. The authors alone are responsible for the content and writing of the paper.

## References

[CIT0001] Tandan R, Bradley WG (1985). Amyotrophic lateral sclerosis: Part 1. Clinical features, pathology, and ethical issues in management. Ann Neurol..

[CIT0002] Mulder DW, Kurland LT, Offord KP, Beard CM (1986). Familial adult motor neuron disease: amyotrophic lateral sclerosis. Neurology..

[CIT0003] Camu W, Khoris J, Moulard B, Salachas F, Briolotti V, Rouleau GA (1999). Genetics of familial ALS and consequences for diagnosis. French ALS Research Group. J Neurol Sci..

[CIT0004] Rosen DR, Siddique T, Patterson D, Figlewicz DA, Sapp P, Hentati A (1993). Mutations in Cu/Zn superoxide dismutase gene are associated with familial amyotrophic lateral sclerosis. Nature..

[CIT0005] Chen YZ, Bennett CL, Huynh HM, Blair IP, Puls I, Irobi J (2004). DNA/RNA helicase gene mutations in a form of juvenile amyotrophic lateral sclerosis (ALS4). Am J Hum Genet..

[CIT0006] Hadano S, Hand CK, Osuga H, Yanagisawa Y, Otomo A, Devon RS (2001). A gene encoding a putative GTPase regulator is mutated in familial amyotrophic lateral sclerosis 2. Nat Genet..

[CIT0007] Nishimura AL, Mitne-Neto M, Silva HC, Richieri-Costa A, Middleton S, Cascio D (2004). A mutation in the vesicle-trafficking protein VAPB causes late-onset spinal muscular atrophy and amyotrophic lateral sclerosis. Am J Hum Genet..

[CIT0008] Yang Y, Hentati A, Deng HX, Dabbagh O, Sasaki T, Hirano M (2001). The gene encoding alsin, a protein with three guanine-nucleotide exchange factor domains, is mutated in a form of recessive amyotrophic lateral sclerosis. Nat Genet..

[CIT0009] Hafezparast M, Klocke R, Ruhrberg C, Marquardt A, Ahmad-Annuar A, Bowen S (2003). Mutations in dynein link motor neuron degeneration to defects in retrograde transport. Science..

[CIT0010] Morita M, Al-Chalabi A, Andersen PM, Hosler B, Sapp P, Englund E (2006). A locus on chromosome 9p confers susceptibility to ALS and frontotemporal dementia. Neurology..

[CIT0011] Sapp PC, Hosler BA, McKenna-Yasek D, Chin W, Gann A, Genise H (2003). Identification of two novel loci for dominantly inherited familial amyotrophic lateral sclerosis. Am J Hum Genet..

[CIT0012] Ruddy DM, Parton MJ, Al-Chalabi A, Lewis CM, Vance C, Smith BN (2003). Two families with familial amyotrophic lateral sclerosis are linked to a novel locus on chromosome 16q. Am J Hum Genet..

[CIT0013] Vance C, Al-Chalabi A, Ruddy D, Smith BN, Hu X, Sreedharan J (2006). Familial amyotrophic lateral sclerosis with frontotemporal dementia is linked to a locus on chromosome 9p13.2-21.3. Brain..

[CIT0014] Graham AJ, Macdonald AM, Hawkes CH (1997). British motor neuron disease twin study. J Neurol Neurosurg Psychiatry..

[CIT0015] Kent WJ, Sugnet CW, Furey TS, Roskin KM, Pringle TH, Zahler AM (2002). The human genome browser at UCSC. Genome Res..

[CIT0016] Aldridge WN (1953). Serum esterases. II. An enzyme hydrolysing diethyl p-nitrophenyl phosphate (E600) and its identity with the A-esterase of mammalian sera. Biochem J..

[CIT0017] Furlong CE, Richter RJ, Seidel SL, Costa LG, Motulsky AG (1989). Spectrophotometric assays for the enzymatic hydrolysis of the active metabolites of chlorpyrifos and parathion by plasma paraoxonase/arylesterase. Anal Biochem..

[CIT0018] Beal MF, Ferrante RJ, Browne SE, Matthews RT, Kowall NW, Brown RH (1997). Increased 3-nitrotyrosine in both sporadic and familial amyotrophic lateral sclerosis. Ann Neurol..

[CIT0019] Cellini E, Tedde A, Bagnoli S, Nacmias B, Piacentini S, Bessi V (2006). Association analysis of the paraoxonase-1 gene with Alzheimer's disease. Neurosci Lett..

[CIT0020] Erlich PM, Lunetta KL, Cupples LA, Huyck M, Green RC, Baldwin CT (2006). Polymorphisms in the PON gene cluster are associated with Alzheimer's disease. Hum Mol Genet..

[CIT0021] Akhmedova SN, Yakimovsky AK, Schwartz EI (2001). Paraoxonase 1 Met-Leu 54 polymorphism is associated with Parkinson's disease. J Neurol Sci..

[CIT0022] Kondo I, Yamamoto M (1998). Genetic polymorphism of paraoxonase 1 (PON1) and susceptibility to Parkinson's disease. Brain Res..

[CIT0023] Dantoine TF, Debord J, Merle L, Lacroix-Ramiandrisoa H, Bourzeix L, Charmes JP (2002). Paraoxonase 1 activity: a new vascular marker of dementia?. Ann N Y Acad Sci..

[CIT0024] Wheeler JG, Keavney BD, Watkins H, Collins R, Danesh J (2004). Four paraoxonase gene polymorphisms in 11,212 cases of coronary heart disease and 12,786 controls: meta-analysis of 43 studies. Lancet..

[CIT0025] Cronin S, Greenway MJ, Prehn JH, Hardiman O (2007). Paraoxonase promoter and intronic variants modify risk of sporadic amyotrophic lateral sclerosis. J Neurol Neurosurg Psychiatry..

[CIT0026] Morahan JM, Yu B, Trent RJ, Pamphlett R (2007). A gene-environment study of the paraoxonase 1 gene and pesticides in amyotrophic lateral sclerosis. Neurotoxicology..

[CIT0027] Saeed M, Siddique N, Hung WY, Usacheva E, Liu E, Sufit RL (2006). Paraoxonase cluster polymorphisms are associated with sporadic ALS. Neurology..

[CIT0028] Slowik A, Tomik B, Wolkow PP, Partyka D, Turaj W, Malecki MT (2006). Paraoxonase gene polymorphisms and sporadic ALS. Neurology..

[CIT0029] Shaw CE, Al-Chalabi A (2006). Susceptibility genes in sporadic ALS: separating the wheat from the chaff by international collaboration. Neurology..

[CIT0030] Brooks BR, Miller RG, Swash M, Munsat TL (2000). El Escorial revisited: revised criteria for the diagnosis of amyotrophic lateral sclerosis. Amyotroph Lateral Scler Other Motor Neuron Disord..

[CIT0031] Carlson CS, Eberle MA, Rieder MJ, Yi Q, Kruglyak L, Nickerson DA (2004). Selecting a maximally informative set of single-nucleotide polymorphisms for association analyses using linkage disequilibrium. Am J Hum Genet..

[CIT0032] Purcell S, Neale B, Todd-Brown K, Thomas L, Ferreira MA, Bender D (2007). PLINK: a tool set for whole-genome association and population-based linkage analyses. Am J Hum Genet..

[CIT0033] A-M Wills, Landers J, Zhang H, Richter R, Caraganis A, Cudkowicz M (2007). Paraoxonase 1 (PON1) organophosphate hydrolysis is not reduced in ALS. Neurology.

[CIT0034] Adkins S, Gan KN, Mody M, La Du BN (1993). Molecular basis for the polymorphic forms of human serum paraoxonase/arylesterase: glutamine or arginine at position 191, for the respective A or B allozymes. Am J Hum Genet..

[CIT0035] Brophy VH, Jampsa RL, Clendenning JB, McKinstry LA, Jarvik GP, Furlong CE (2001). Effects of 5′ regulatory-region polymorphisms on paraoxonase-gene (PON1) expression. Am J Hum Genet..

[CIT0036] Garin MC, James RW, Dussoix P, Blanche H, Passa P, Froguel P (1997). Paraoxonase polymorphism Met-Leu54 is associated with modified serum concentrations of the enzyme. A possible link between the paraoxonase gene and increased risk of cardiovascular disease in diabetes. J Clin Invest..

[CIT0037] McGuire V, Longstreth WT, Nelson LM, Koepsell TD, Checkoway H, Morgan MS (1997). Occupational exposures and amyotrophic lateral sclerosis. A population-based case-control study. Am J Epidemiol..

[CIT0038] Horner RD, Kamins KG, Feussner JR, Grambow SC, Hoff-Lindquist J, Harati Y (2003). Occurrence of amyotrophic lateral sclerosis among Gulf War veterans. Neurology..

[CIT0039] Weisskopf MG, O'Reilly EJ, McCullough ML, Calle EE, Thun MJ, Cudkowicz M (2005). Prospective study of military service and mortality from ALS. Neurology..

[CIT0040] Costa LG, Vitalone A, Cole TB, Furlong CE (2005). Modulation of paraoxonase (PON1) activity. Biochem Pharmacol..

[CIT0041] Mackness MI, Arrol S, Durrington PN (1991). Paraoxonase prevents accumulation of lipoperoxides in low-density lipoprotein. FEBS Lett..

[CIT0042] Aviram M, Rosenblat M, Bisgaier CL, Newton RS, Primo-Parmo SL, La Du BN (1998). Paraoxonase inhibits high-density lipoprotein oxidation and preserves its functions. A possible peroxidative role for paraoxonase. J Clin Invest..

[CIT0043] Oda MN, Bielicki JK, Ho TT, Berger T, Rubin EM, Forte TM (2002). Paraoxonase 1 overexpression in mice and its effect on high-density lipoproteins. Biochem Biophys Res Commun..

[CIT0044] Reynolds A, Laurie C, Mosley RL, Gendelman HE (2007). Oxidative stress and the pathogenesis of neurodegenerative disorders. Int Rev Neurobiol..

[CIT0045] Veldink JH, Kalmijn S, Groeneveld GJ, Wunderink W, Koster A, de Vries JH (2007). Intake of polyunsaturated fatty acids and vitamin E reduces the risk of developing amyotrophic lateral sclerosis. J Neurol Neurosurg Psychiatry..

[CIT0046] A haplotype map of the human genome (2005). Nature.

[CIT0047] Dunckley T, Huentelman MJ, Craig DW, Pearson JV, Szelinger S, Joshipura K (2007). Whole-genome analysis of sporadic amyotrophic lateral sclerosis. N Engl J Med..

[CIT0048] Nishio E, Watanabe Y (1997). Cigarette smoke extract inhibits plasma paraoxonase activity by modification of the enzyme's free thiols. Biochem Biophys Res Commun..

[CIT0049] Shih DM, Gu L, Hama S, Xia YR, Navab M, Fogelman AM (1996). Genetic-dietary regulation of serum paraoxonase expression and its role in atherogenesis in a mouse model. J Clin Invest..

[CIT0050] Gouedard C, Koum-Besson N, Barouki R, Morel Y (2003). Opposite regulation of the human paraoxonase-1 gene PON-1 by fenofibrate and statins. Mol Pharmacol..

[CIT0051] Sozmen EY, Mackness B, Sozmen B, Durrington P, Girgin FK, Aslan L (2002). Effect of organophosphate intoxication on human serum paraoxonase. Hum Exp Toxicol..

[CIT0052] Cronin S, Berger S, Ding J, Schymick JC, Washecka N, Hernandez DG (2008). A genome-wide association study of sporadic ALS in a homogenous Irish population. Hum Mol Genet..

[CIT0053] Schymick JC, Scholz SW, Fung HC, Britton A, Arepalli S, Gibbs JR (2007). Genome-wide genotyping in amyotrophic lateral sclerosis and neurologically normal controls: first stage analysis and public release of data. Lancet Neurol..

[CIT0054] van Es MA, van Vught PW, Blauw HM, Franke L, Saris CG, Andersen PM (2007). ITPR2 as a susceptibility gene in sporadic amyotrophic lateral sclerosis: a genome-wide association study. Lancet Neurol..

[CIT0055] van Es MA, van Vught PW, Blauw HM, Franke L, Saris CG, van den Bosch L (2008). Genetic variation in DPP6 is associated with susceptibility to amyotrophic lateral sclerosis. Nat Genet..

